# Baseline angina burden predicts quality of life and functional improvement in patients with viable myocardium treated for chronic total occlusion

**DOI:** 10.1007/s10554-023-02916-9

**Published:** 2023-07-12

**Authors:** Johannes Kersten, Vanessa Maisenbacher, Pauline Fengel, Yvonne Werner, Carsten Hackenbroch, Meinrad Beer, Sören Westphal, Peter Bernhardt

**Affiliations:** 1 Department for Internal Medicine, Deputy for Sports and Rehabilitation Medicine, University Hospital Ulm, University of Ulm, Leimgrubenweg 14, 89071 Ulm, Deutschland; 2 Heart Clinic Ulm, Ulm, Germany; 3 Department of Radiology, Armed Forces Military Hospital Ulm, Ulm, Germany; 4 Department of Diagnostic and Interventional Radiology, University of Ulm, Ulm, Germany

**Keywords:** Chronic total occlusion, Cardiac magnetic resonance imaging, Myocardial revascularisation, Outcomes and prognosis, Quality of life

## Abstract

Chronic total occlusion (CTO) is a common finding in patients with known or suspected coronary artery disease and has a distinctive role in these patients’ quality of life. However, there is still a lack of evidence of correct patient selection for percutaneous coronary intervention (PCI). From July 2017 to August 2020, 68 patients with successful PCI of a CTO and previous evidence of viability for PCI by cardiovascular magnetic resonance imaging (CMR) were prospectively included in this single-centre observational study. Of these patients, 62 underwent follow-up CMR, and 56 underwent surveys using the Seattle Angina Questionnaire before PCI and 3, 12 and 24 months after PCI. The CMR results were assessed for volumetric, functional and deformation parameters. From the baseline to the follow-up, there was a significant reduction in the left ventricular volumes (all *p* < 0.001) and an increase in the left ventricular ejection fraction (57.6 ± 11.6% vs. 60.3 ± 9.4%, *p* = 0.006). Among the deformation parameters, only the left ventricular radial strain showed significant improvement. The SAQ showed an early improvement that emphasised angina stability and frequency as well as a summary score, which persisted after 24 months. A low SAQ summary score before PCI was the best predictive factor of good clinical improvement thereafter. Improvements in myocardial function and quality of life can be achieved with PCI of a CTO. Patient selection for PCI should be performed primarily among relevantly symptomatic patients when evidence of viability for PCI is present. The SAQ can help guide such patient selection.

***Trial registration*** ISRCTN, identifier: ISRCTN33203221. Retrospectively registered on 01.04.2020. https://www.isrctn.com/ISRCTN33203221

## Introduction

Chronic total occlusion (CTO) is a common finding in patients with stable angina pectoris [1]. CTO has been described as inversely associated with event-free survival in coronary artery disease. Registries and meta-analyses have shown improved clinical symptoms and functional parameters in revascularised CTO [2–4]. Nevertheless, the presence of a CTO is the greatest predictor of incomplete revascularisation. This is due to, on the one hand, the low evidence of the prognostic benefit, and on the other hand, the higher rate of major adverse cardiovascular events, the longer fluoroscopy time and the higher contrast agent requirement.

Current ESC/EACTS guidelines on myocardial revascularisation recommend non-invasive diagnostic assessment of patients’ myocardial viability for percutaneous coronary intervention (PCI) prior to an attempt at revascularisation by PCI [5]. However, data on correct patient selection based on non-invasive imaging are lacking [6]. The EURO-CTO study has already shown the symptomatic benefits of CTO-PCI [7], but which patients are likelier to benefit symptomatically from revascularisation remains a matter of debate. However, it is the symptomatology as a soft end point that is of crucial importance for the quality of life of our patients.

There are indications that cardiac magnetic resonance imaging (CMR) detection of a sufficiently large perfusion deficit and of the patient’s viability for PCI in the concerned coronary territory is likeliest to be accompanied by good symptom relief after revascularisation [3, 7]. CMR offers high diagnostic accuracy for the detection of myocardial ischemia and of the patient’s viability for PCI. As the gold standard for determining cardiac volumetry and function, it also offers the possibility of deformation analysis using feature tracking in the post-processing. Due to its multi-modality approach, it is an ideal tool for pre-diagnosis of CTO in patients.

This study attempts to answer the following questions:

1. How do the symptomatology and functional parameters of cardiac function change longitudinally after CTO-PCI?
2. Are there patient groups that particularly benefit clinically from CTO-PCI?

## Methods

### Study design

This study is a prospective observational trial of CTO patients in a single high-volume outpatient cardiological care centre. A written or oral survey using the Seattle Angina Questionnaire (SAQ) was conducted at baseline and after 3, 12, and 24 months to assess the patients’ clinical status. CMR-scans were performed before and after CTO-PCI to evaluate viability of the affected myocardium, but also functional parameters and valve diseases in general.

### Participants

All the patients at the centre who demonstrated angiography-proven CTO from July 2017 to August 2020 were screened for participation in this study. The exclusion criteria were myocardial infarction within the last three months; unstable angina; contraindications for CMR, gadolinium-based contrast agents or intravenous administration of adenosine; impaired renal function (glomerular filtration rate < 30 ml/min); and inability to give their written informed consent. The exclusion criterion for interventional revascularisation was transmural scarring of the CTO-related territory. This was defined as late gadolinium enhancement imaging (LGE) of more than 70% in three or more CTO-related segments. The included patients each gave their written informed consent to participate in this study before they were enrolled in it.

**Fig. 1:**
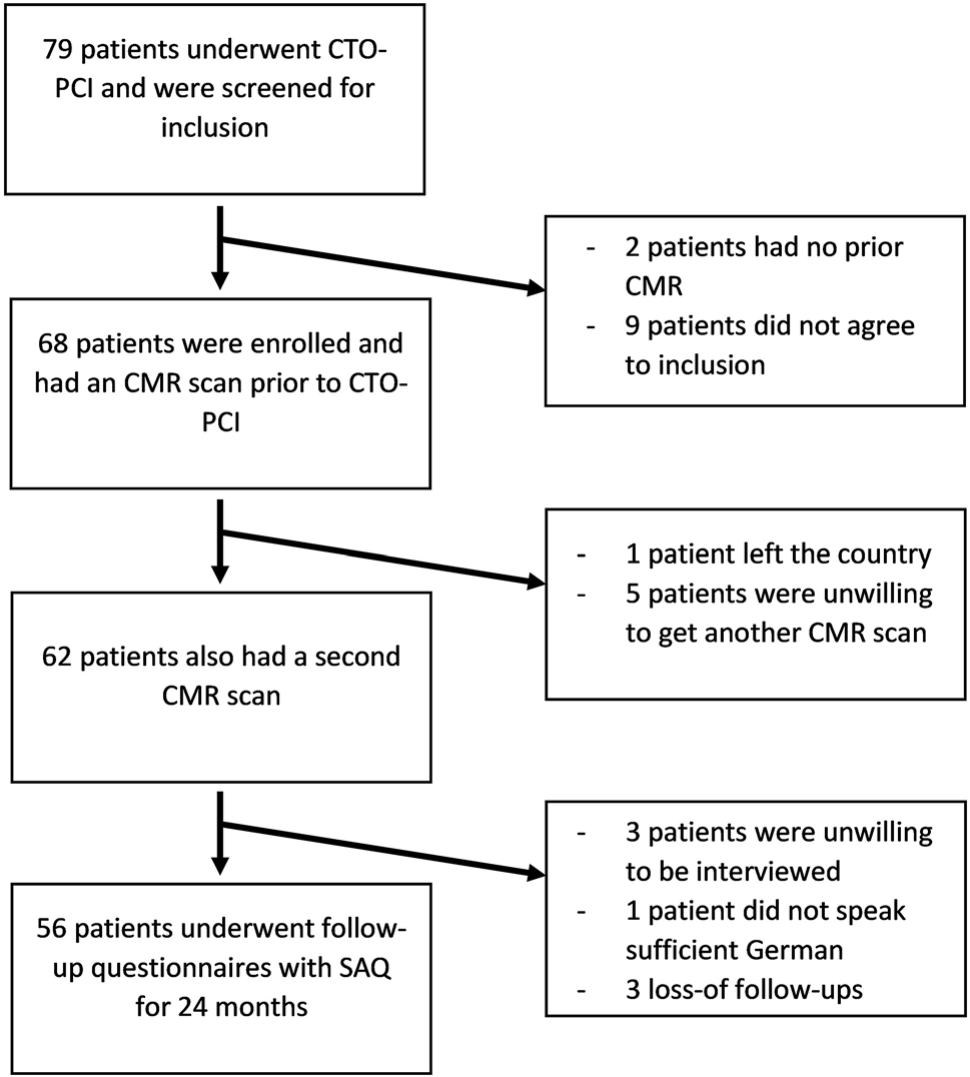
Patient enrollment. *CMR* cardiovascular magnetic resonance, *CTO* chronic total occlusion, *PCI* percutaneous coronary intervention, *SAQ* Settle Angina Questionnaire

**Fig. 2:**
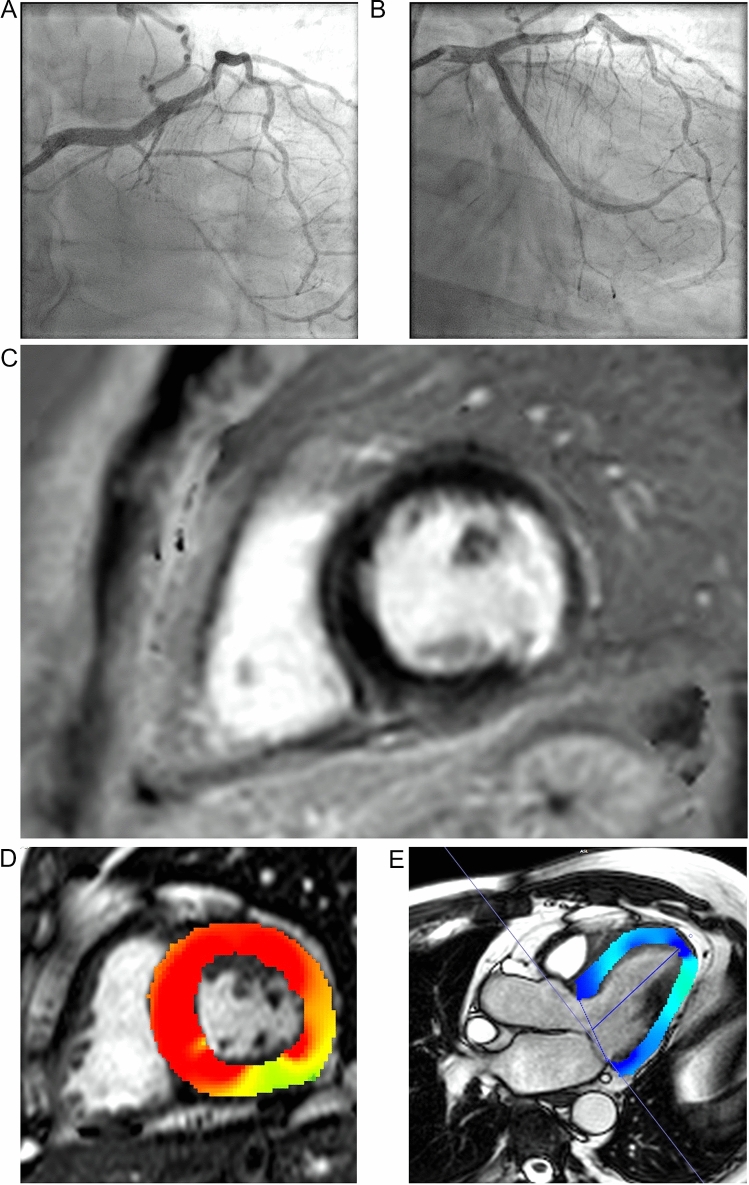
Example of a patient with chronic total occlusion of the left circumflex (LCX). **A** Angiography before and **B** after percutaneous coronary intervention. The cardiac magnetic resonance imaging prior to the revascularisation demonstrated **C** non-transmural scarring in the late-gadolinium enhancement (LGE) image and **D** hypokinesia of the lateral or posterolateral wall, as seen in the colour-encoded images of the radial strain **D** and **E** longitudinal strain

Sixty-eight patients were enrolled in the study. The patient enrolment is visualised in Fig. 1. The patients were 66.7 ± 8.1 years old, and most of them were men (75%) with a high prevalence of cardiovascular risk factors, as shown in Table 1. Eight patients (11.8%) had prior failed CTO-PCI. The mean J-CTO score was 2.7 ± 1.3, with a collateral (Rentrop) score of 2.0 ± 0.7. The right coronary artery (RCA) was most frequently affected by a CTO (RCA, 63.2%), followed by the left anterior descending artery (LAD, 22.1%) and the left circumflex artery (LCX, 14.7%). The revascularisations were performed by a single operator (P.B.) predominantly in antegrade technique, with 9 retrograde procedures (13.2%). An example of a patient with a CTO of the LCX is shown in Fig. 2. Adverse events occurred in 15 out of 68 patients (22.1%) included in the study. Access site events, such as hematoma or pseudoaneurysm, were observed in 7 out of 68 patients (10.3%). Complications related to the coronary arteries were observed in 5 out of 68 patients (8.8%), including 4 cases of unplanned dissections requiring stenting and one instance of a broken guidewire. Additionally, there was one pericardial effusion necessitating pericardiocentesis.

**Table 1:** Patient characteristics (*n* = 68)

Characteristic	Value
Age, *y*	66.7 ± 8.1
Women, *n* (%)	17 (25.0)
Body mass index, kg/m^2^	29.1 ± 4.6
Hypertension, *n* (%)	58 (85.3)
Diabetes mellitus, *n* (%)	22 (32.4)
Dyslipidaemia, *n* (%)	60 (88.2)
Smokers, *n* (%)	12 (17.6) [Ex-smokers 12 (17.6)]
Family history of CAD	22 (32.4)
Patient history	
Previous percutaneous coronary intervention, *n* (%)	45 (66.2)
Previous coronary artery bypass surgery, *n* (%)	3 (4.4)
Vessel status	
1-vessel disease, *n* (%)	18 (26.5)
2-vessel disease, *n* (%)	18 (26.5)
3-vessel disease, *n* (%)	32 (47.1)
Medication	
Betablockers, *n* (%)	53 (77.9)
ACE inhibitors/AT1 receptor blockers, *n* (%)	43 (63.2)
Mineralocorticoid receptor blockers, *n* (%)	8 (11.8)
Statins, *n* (%)	59 (86.8)
Ezetimib, *n* (%)	10 (14.7)
	11 (16.2)
CTO-related coronary artery	
Left anterior descending	15 (22.1)
Left circumflex	10 (14.7)
Right coronary artery	43 (63.2)
J-CTO score	2.7 ± 1.3
PROGRESS-CTO score	1.5 ± 1.1
EURO-CTO score	2.6 ± 1.3
Rentrop score	2.0 ± 0.7

*CAD* coronary artery disease, *CTO* chronic total occlusion

### The Seattle Angina Questionnaire (SAQ)

SAQ is a well-established and validated instrument for evaluating the functional and emotional status of patients with coronary artery disease [8]. We used its German version for this study. The SAQ has 17 questions on a five-point descriptive scale. They ask about physical limitations, angina stability, angina frequency, treatment satisfaction and disease perception. Each of these five subjects was measured and transformed to a scale of 0 (lowest) to 100 (highest). An alteration of 10 points was assumed to have been clinically relevant. To simplify the assessment of the course of functional complaints, a summary score was formed from the three subscales: physical limitations, angina stability and angina frequency.

### Cardiac magnetic resonance

CMR was performed in all the patients using either a 1.5 T or 3.0 T scanner with a 32-channel phased-array cardiac surface coil. A steady-state, free-precision cine sequence in three long-axis views (2-, 3- and 4-chamber view orientations) and contiguous short-axis orientations was obtained for volumetric and functional analyses.

For LGE, gadoterate meglumine (Dotarem®, Guerbet, Villepinte, France) was administered intravenously at a cumulative dose of 0.2 mmol/kg of body weight. To adjust the individual inversion time, a look-locker sequence in a short-axis orientation was performed 10 min thereafter. An inversion-recovery gradient-echo sequence for the evaluation of LGE was acquired in the same orientations as the cine image.

All the images were evaluated by two experienced examiners (J.K. and P.F.) in consensus using an established software (cvi42, Circle, Calgary, Canada). In cases of disagreement, a third investigator (P.B.) was consulted to make a final decision. They were blinded to the clinical data, SAQ results and time of image acquisition (before or after the PCI). The cine images were analysed for the left and right ventricular volumes and the myocardial masses. The ejection fractions were calculated correspondingly. Using post-processing feature tracking, the strain parameters were obtained. The LGE images were evaluated for the presence of myocardial scarring. A transmurality of more than 70% in three or more CTO-related segments was considered non-viable for PCI, so CTO recanalisation was not performed.

### Statistical analysis

For the descriptive analysis, the continuous variables were expressed as means ± standard deviations, and the categorical values were expressed as numbers and percentages. All the data were normally distributed according to graphical analysis or the Kolmogorov–Smirnov test. Two ordinally scaled values were comparatively analysed with a two-tailed Students’ t-test; and to compare more than two values, analysis of variance (ANOVA) was correspondingly used. The categorical variables were compared using the chi-square test. A *p*-value of < 0.05 was considered statistically significant. The statistical analysis was performed using IBM SPSS Statistics 26 (IBM, Armonk, NY, USA).

## Results

### Changes in the cardiac function

Of the total cohort, 62 patients also underwent CMR at follow-up. This took place for a mean of 91 ± 32 days after the CTO-PCI. At the baseline, the left and right ventricular functions were normal on average, as shown in Table 2. There was slight left ventricular dilatation. At the follow-up, there were statistically significant reductions in the left ventricular end-diastolic and end-systolic volumes (156.5 ± 42.0 ml vs. 145.3 ± 34.7 ml, *p* < 0.001 and 69.0 ± 34.8 ml vs. 59.2 ± 23.8 ml, *p* < 0.001) with improvement in the left ventricular ejection fraction (57.6 ± 11.6% vs. 60.3 ± 9.4%, *p* = 0.006). The right ventricular volumes did not change significantly from the baseline to the follow-up.

**Table 2:** Cardiac magnetic resonance imaging results of all the patients who underwent imaging at the baseline and the follow-up (*n* = 62)

Value	Baseline	Follow-up	*p*-value
Volumetry			
LV end-diastolic volume, ml	156.5 ± 42.0	145.3 ± 34.7	< .001
LV end-diastolic volume indexed, ml/m^2^	77.6 ± 19.3	72.1 ± 15.7	< .001
LV end-systolic volume, ml	69.0 ± 34.8	59.2 ± 23.8	< .001
LV stroke volume, ml	87.5 ± 20.6	86.4 ± 20.1	0.580
LV ejection fraction, %	57.6 ± 11.6	60.3 ± 9.4	0.006
LV mass, g	113.2 ± 25.1	111.4 ± 23.4	0.369
RV end-diastolic volume, ml	142.1 ± 39.8	142.2 ± 35.5	0.998
RV end-diastolic volume indexed, ml/m^2^	70.0 ± 16.9	70.3 ± 15.6	0.864
RV ejection fraction, %	47.1 ± 9.2	48.8 ± 10.1	0.203
Deformation parameters			
LV global radial strain (%)	26.2 ± 8.3	27.7 ± 8.3	0.021
LV global circumferential strain (%)	− 16.0 ± 3.6	− 16.0 ± 5.8	0.983
LV global longitudinal strain (%)	− 12.9 ± 3.0	− 13.4 ± 2.9	0.121
RV global longitudinal strain (%)	− 21.1 ± 4.1	− 20.9 ± 5.4	0.838
LV peak systolic radial strain rate (/s)	1.3 ± 0.4	1.4 ± 0.4	0.008
LV peak systolic circumferential strain rate (/s)	− 0.8 ± 0.2	− 0.8 ± 0.2	0.012
LV peak systolic longitudinal strain rate (/s)	− 0.7 ± 0.3	− 0.7 ± 0.3	0.841
RV peak systolic longitudinal strain rate (/s)	− 1.3 ± 0.6	− 1.1 ± 0.7	0.146
LV peak diastolic radial strain rate (/s)	− 1.3 ± 0.6	− 1.3 ± 0.6	0.259
LV peak diastolic circumferential strain rate (/s)	0.7 ± 0.2	0.8 ± 0.2	0.233
LV peak diastolic longitudinal strain rate (/s)	0.6 ± 0.2	0.7 ± 0.2	0.455
RV peak diastolic longitudinal strain rate (/s)	1.1 ± 0.6	1.1 ± 0.5	0.453

*LV* left ventricular, *RV* right ventricular

In contrast to the significant improvements in the left ventricular volumetry, there was hardly any statistically significant change in the deformation parameters. Significant improvements were seen only in the left ventricular global radial strain (26.2 ± 8.3% vs. 27.7 ± 8.3%, *p* = 0.021) and the left ventricular radial and circumferential strains (both, *p* < 0.05). The remaining volumetric and deformation parameters can be found in Table 2.

### Changes in the SAQ

**Fig. 3:**
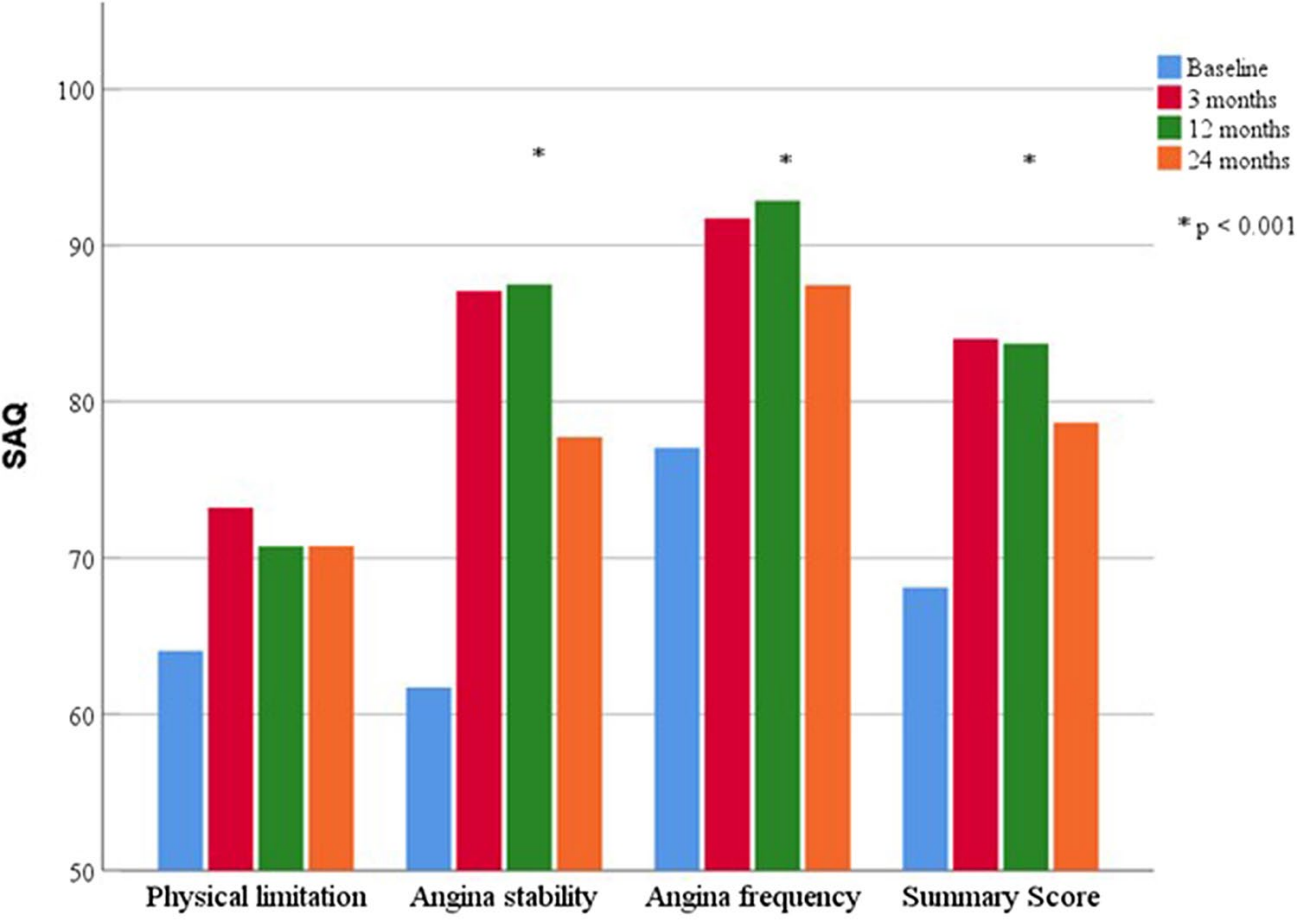
Seattle Angina Questionnaire before and 3, 12 and 24 months after percutaneous coronary intervention of a chronic total occlusion

Of the included patients, 56 participated in the follow-up interviews using SAQ. The summary score for the three functional subscales—physical limitation, angina stability and angina frequency—improved significantly (*p* < 0.001) in the Bonferroni post-hoc test compared to all the follow-up time points. Thus, at the first follow-up, there was an improvement from 69 ± 19 to 84 ± 14 (*p* < 0.001), which remained basically stable until the 24-month follow-up (79 ± 19, *p* = 0.006). This was predominantly due to the statistically significant improvements in the angina stability and frequency. The physical limitations did not change relevantly from the baseline to the follow-up interviews (*p* = 0.250), as shown in Fig. 3. The treatment satisfaction and disease acceptance also improved statistically significantly (*p* = 0.040 and *p* < 0.001, respectively). Overall, all the parameters improved early after the revascularisation, with a discrete decline over time, as shown in Table 3.

**Table 3:** Changes in the scales of the Seattle Angina Questionnaire for all the patients who answered the follow-up questionnaires (*n* = 56)

Value	Baseline	After 3 months	After 12 months	After 24 months	*p*-value
Physical limitation	66 ± 19	73 ± 20	71 ± 24	71 ± 21	0.250
Angina stability	62 ± 26	87 ± 21	88 ± 19	78 ± 26	< .001
Angina frequency	77 ± 25	92 ± 13	93 ± 13	87 ± 22	< .001
Treatment satisfaction	81 ± 18	81 ± 17	79 ± 16	73 ± 18	0.040
Disease perception	50 ± 28	70 ± 23	77 ± 21	75 ± 21	< .001

### Prediction of symptomatic improvement

All the patients were divided into three groups using the summary SAQ score composed of the arithmetic means for the physical limitation, angina stability and angina frequency subscales. An improvement of more than 10 points in the SAQ three months after the CTO-PCI was presumed to be a good clinical response, and the patient was categorised in the ‘responders’ group. If the SAQ was enhanced only from 0 to 10 points, the response was low, and the patient was categorised in the ‘low-responders’ group. If there was no difference from the baseline to the follow-up or if the SAQ decreased, the patient was categorised in the ‘non-responder’ group. The responder group was the largest, with 34 patients (60.7%), followed by the low-responders group (*n* = 17, 30.4%) and the non-responders group (*n* = 5, 8.9%).

As shown in Table 4, there were no relevant differences in the baseline characteristics, especially with regard to age, sex, cardiovascular risk factors and medication (all *p* > 0.05). The angiographic characteristics likewise did not significantly differ between the responders, low-responders and non-responders groups. In the baseline CMR, there was, at most, a tendency towards lower-left ventricular volumes and discretely better left ventricular function in the non-responders group, although no parameter fell below the significant level. This was also the case for the deformation parameters, as shown in Table 4.

**Table 4:** Patient and angiographic characteristics, as well as cardiac magnetic resonance imaging and Seattle Angina Questionnaire (SAQ) results at the baseline in dependence on the clinical result three months after percutaneous revascularisation of a chronic total occlusion

Value	Non-responder (*n* = 5)	Low-responder (*n* = 17)	Responder (*n* = 34)	*p*-value
Age, *y*	66.4 ± 12.0	65.5 ± 7.3	67.0 ± 7.5	0.827
Women, *n* (%)	3 (60.0)	2 (11.8)	10 (29.4)	0.087
Body mass index, kg/m^2^	31.5 ± 3.7	28.3 ± 5.1	28.5 ± 4.2	0.347
Hypertension, *n* (%)	4 (80.0)	14 (82.4)	31 (91.2)	0.580
Diabetes mellitus, *n* (%)	0 (0.0)	3 (17.6)	13 (38.2)	0.103
Dyslipidaemia, *n* (%)	5 (100)	17 (100)	30 (88.2)	0.123
Smokers, *n* (%)	1 (20.0)	6 (35.3)	12 (35.3)	0.789
Family history of CAD	2 (40.0)	9 (52.9)	9 (26.5)	0.174
Patient history				
Previous percutaneous coronary intervention, *n* (%)	4 (80.0)	12 (70.6)	21 (61.8)	0.648
Previous coronary artery bypass surgery, *n* (%)	0 (0.0)	1 (5.9)	2 (5.9)	0.856
Vessel status				0.561
1-vessel disease, *n* (%)	1 (20.0)	4 (23.5)	11 (32.4)	
2-vessel disease, *n* (%)	1 (20.0)	7 (41.2)	7 (20.6)	
3-vessel disease, *n* (%)	3 (60.0)	6 (35.3)	16 (47.1)	
Medication at the time of inclusion				
Betablockers, *n* (%)	4 (80.0)	13 (76.5)	27 (79.4)	0.968
ACE inhibitors/AT1 receptor blockers, *n* (%)	2 (40.0)	11 (64.7)	23 (67.7)	0.621
Mineralocorticoid receptor blockers, *n* (%)	0 (0.0)	1 (5.9)	5 (14.7)	0.454
Statins, *n* (%)	5 (100)	14 (82.4)	32 (94.1)	0.291
Ezetimib, *n* (%)	1 (20.0)	2 (11.8)	5 (14.7)	0.893
CTO-related coronary artery				0.588
Left anterior descending	1 (20.0)	3 (17.6)	8 (23.5)	
Left circumflex	0 (0.0)	1 (5.9)	6 (17.6)	
Right coronary artery	4 (80.0)	13 (76.5)	20 (58.8)	
J-CTO score	2.6 ± 1.1	2.4 ± 1.2	2.7 ± 1.5	0.652
PROGRESS-CTO score	2.2 ± 0.8	1.1 ± 1.2	1.4 ± 1.0	0.094
EURO-CTO score	2.8 ± 1.5	2.3 ± 1.1	2.6 ± 1.4	0.704
Rentrop score	1.6 ± 0.5	2.3 ± 0.6	1.9 ± 0.7	0.049
Volumetry at the baseline				
LV end-diastolic volume, *ml*	136.9 ± 37.2	161.3 ± 35.6	154.7 ± 44.5	0.513
LV end-diastolic volume indexed, ml/m^2^	68.9 ± 15.0	79.3 ± 16.1	77.7 ± 22.4	0.595
LV end-systolic volume, ml	50.0 ± 21.3	65.3 ± 27.0	69.8 ± 37.5	0.465
LV stroke volume, ml	86.9 ± 19.2	96.0 ± 26.5	84.9 ± 16.2	0.580
LV ejection fraction, %	64.4 ± 7.1	59.9 ± 10.9	57.2 ± 11.5	0.354
LV mass, g	92.9 ± 21.5	120.2 ± 24.0	113.2 ± 22.7	0.074
RV end-diastolic volume, ml	131.4 ± 44.6	157.0 ± 32.9	136.5 ± 41.9	0.188
RV end-diastolic volume indexed, ml/m^2^	65.1 ± 15.0	77.1 ± 14.7	67.8 ± 18.3	0.150
RV ejection fraction, %	44.8 ± 11.3	49.0 ± 9.8	46.5 ± 8.5	0.560
Deformation parameters at the baseline				
LV global radial strain (%)	31.6 ± 8.5	26.2 ± 9.6	26.4 ± 7.3	0.390
LV global circumferential strain (%)	-18.4 ± 3.7	-15.8 ± 3.8	-16.2 ± 3.1	0.346
LV global longitudinal strain (%)	-14.2 ± 2.0	-12.4 ± 2.8	-13.2 ± 2.9	0.423
RV global longitudinal strain (%)	-21.8 ± 5.5	-20.2 ± 4.2	-21.7 ± 3.9	0.469
SAQ at the baseline				
Summary score	88.4 ± 7.0	79.2 ± 14.6	56.9 ± 17.6	< .001
Physical limitation	71.2 ± 14.0	72.9 ± 18.8	60.8 ± 19.8	0.088
Angina stability	100 ± 0.0	73.5 ± 27.2	51.5 ± 20.4	< .001
Angina frequency	94.0 ± 13.4	91.2 ± 14.1	67.4 ± 26.1	.001
Treatment satisfaction	83.4 ± 12.6	76.6 ± 19.5	80.6 ± 19.4	0.702
Disease perception	41.6 ± 15.5	60.9 ± 28.6	45.7 ± 28.3	0.151

The patients were divided according to their SAQ summary score into the non-responders group (with a decrease in SAQ), the low-responders group (with a stable SAQ or an increase of less than 10 points) and the responders group (with an increase of SAQ 10 points or more)

*CAD* coronary artery disease, *CTO* chronic total occlusion, *LV* left ventricular, *RV* right ventricular

The symptomatology at the baseline assessed by the SAQ was inverse to the clinical improvement three months after the CTO-PCI. Thus, compared to the low-responders and responders groups, the non-responders group had a significantly better SAQ summary score at the baseline (88.4 ± 7.0 vs. 79.2 ± 14.6 vs. 56.9 ± 17.6, *p* < 0.001). This was again mainly due to differences in the angina stability (100 ± 0.0 vs. 73.5 ± 27.2 vs. 51.5 ± 20.4, *p* < 0.001) and the angina frequency (94.0 ± 13.4 vs. 91.2 ± 14.1 vs. 67.4 ± 26.1, *p* = 0.001). There were no significant differences in the physical limitation, treatment satisfaction and disease perception at the baseline.

**Fig. 4:**
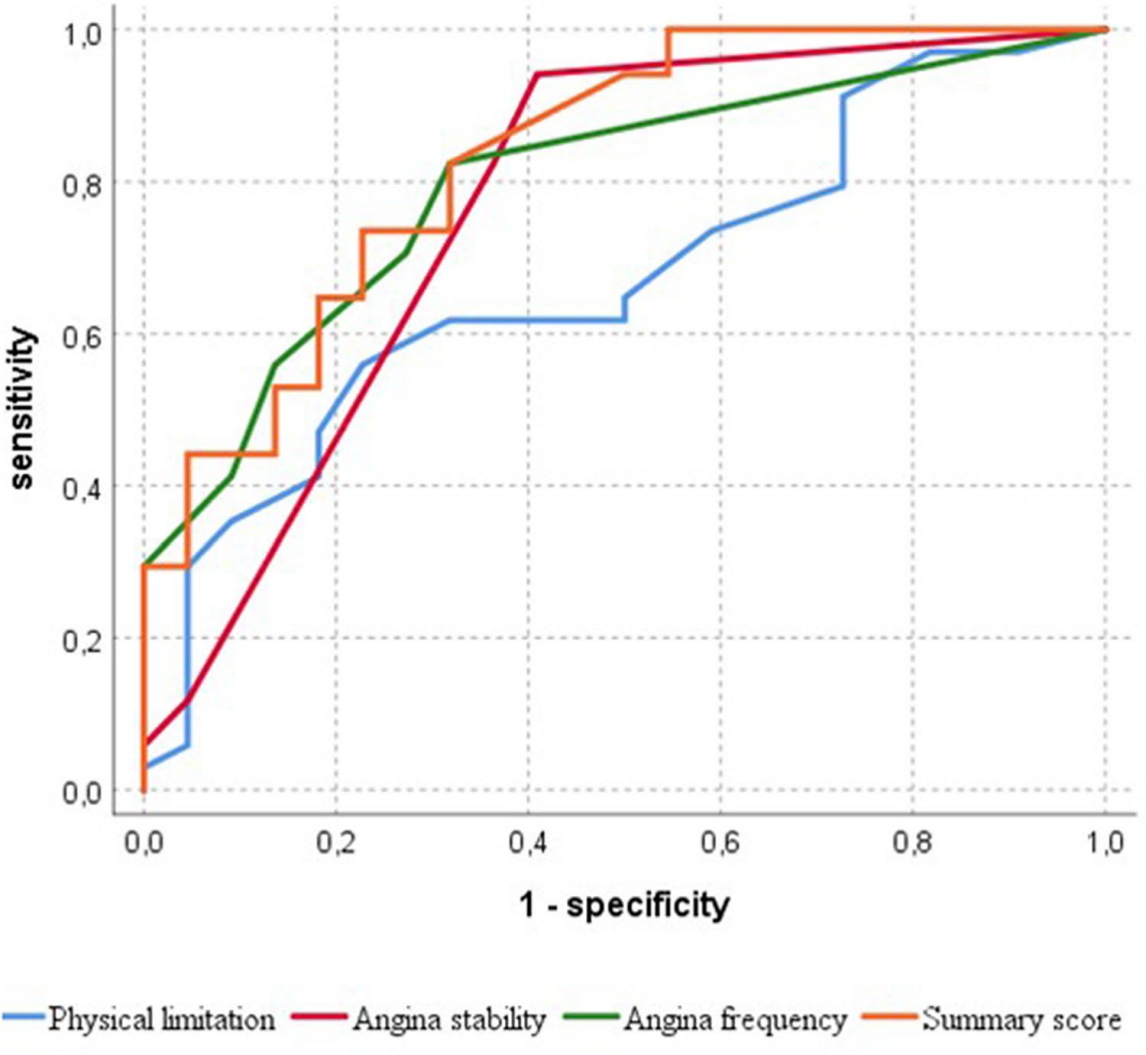
Receiver operating characteristics (ROC) curve of the accuracy of the baseline Seattle Angina Questionnaire (SAQ) values in predicting the clinical outcome of patients with interventional recanalisation of a chronic total occlusion. The lower baseline values at the baseline predicted a higher benefit from coronary intervention. The best values were obtained by the summary score with an area under the curve (AUC) of 0.83 [95% confidence interval (CI) 0.72–0.94]. Accuracy was lower for physical limitation (AUC 0.67, 95% CI 0.53–0.81), angina stability (AUC 0.77, 95% CI 0.63–0.91) and angina frequency (AUC 0.80, 95% CI 0.68–0.91)

In the additional receiver operating characteristics (ROC) analysis, the SAQ summary score at the baseline performed excellently in predicting clinical response to the CTO-PCI, with an area under the curve (AUC) of 0.83, which was higher than that of the physical limitation subscale (AUC 0.67), the angina stability subscale (AUC 0.77) and the angina frequency subscale (AUC 0.80). The ROC curves are shown in Fig. 4. Had a cut-off value of 85 been used for the summary score, all the final responders would have undergone revascularisation. However, PCI would have been avoided in 10 ultimate low- or non-responders (17.9% of all the procedures). This cut-off value had a positive predictive value of 73.9%.

## Discussion

The main findings of this study are as follows. (1) Positive remodelling after CTO-PCI was observed in the patients with viable myocardia. (2) Significant anginal symptom improvement after revascularisation was seen. (3) The clinical responders to the CTO-PCI were primarily patients with pre-existing significant angina. Neither the baseline characteristics nor the initial cardiac volumetry or function had a significant impact on the clinical outcomes with regard to quality of life.

The left ventricular end-diastolic and end-systolic volumes decreased by an average of 11.2 ml and 9.8 ml, respectively. In addition, the ejection fraction improved by an average of 2.7%. Smaller studies in 27 and 32 patients, respectively, showed comparable results [7, 9]. In a study by Galassi et al., the increase in the left ventricular ejection fraction was up to 12.5%, which was more pronounced the lower the baseline ejection fraction [10].

Surprisingly, the volumetric markers of the myocardial function were more markedly affected by revascularisation than were the deformation parameters. Only the radial strain showed significant improvements during the follow-up; the longitudinal and circumferential strains showed trends at best. In ischemic wall motion abnormalities, one would expect a change from the endocardial to epicardial layer. Since the endocardially located myocardial fibres have essentially a longitudinal-oblique orientation, a reduction mainly of the longitudinal strain in the context of myocardial ischemia is to be expected [11]. It is possible that these internal muscle fibres are undergoing fibrotic remodelling, and therefore, little or no recovery can be expected in the longitudinal direction. This assumption was supported in a study by Zhang et al., in which 55 patients who were examined via CMR feature tracking after CTO-PCI showed improvement only in their radial and circumferential strain but not in their longitudinal strain [12]. The effect of revascularisation on the strain parameters was more pronounced the lower the left ventricular ejection fraction was before the procedure. In this study, only 16/68 patients (23.5%) had an ejection fraction below 50% before the CTO-PCI, which might have contributed to the low degree of improvement in the deformation parameters.

The concept of improved myocardial contraction in vital but formerly inferiorly perfused areas is called *hibernation* and was recently challenged by the results of the ‘Percutaneous Revascularization for Ischemic Left Ventricular Dysfunction’ (REVIVED-BCIS2) trial [13]. In that trial, 700 patients with ischemic cardiomyopathy, impaired left ventricular function and imaging evidence of viability were randomised 1:1 to either interventional revascularisation or optimal medical therapy (OMT). There was no significant difference in the ejection fraction between the intervention and control groups at either 6 or 12 months. Considering only the patients with nearly complete revascularisation (anatomical revascularisation index > 80%), there was at least a trend in the primary end point (death from any cause or hospitalisation for heart failure) towards complete revascularisation. The incidence of CTO or CTO-PCI was not reported, although historically, they have been the most common factors of incomplete revascularisation.

In a previously unpublished post-hoc analysis of the ‘Initial Invasive or Conservative Strategy for Stable Coronary Disease’ (ISCHEMIA) trial presented at the 2021 ACC Congress, anatomically complete revascularisation was associated with improvement in the primary end point (cardiovascular death; myocardial infarction; or hospitalisation for cardiac arrest, heart failure or unstable angina), which was not previously demonstrated in the per-protocol analysis [14]. Unfortunately, these data regarding complete revascularization have not been published to date. A smaller study conducted on diabetic patients demonstrated significant differences in terms of mortality and major adverse cardiovascular events between patients who underwent complete revascularization and those who did not [15].

Clinical improvement of patients after CTO-PCI in SAQ has already been demonstrated in other trials[7, 16, 17]. In this study, because of a relatively long follow-up period with repeated administration of interim questionnaires, it was found that the patients’ symptoms deteriorated again over time. Whether this was due to the chronic progression of coronary artery disease, to general ageing processes or to isolated cases of late lesion failure could not be assessed. It is important to acknowledge the potential placebo effect resulting from the invasive procedure itself, as indicated by the findings of the "Percutaneous coronary intervention in stable angina"-trial (ORBITA) [18, 19]. This also may diminish over time. Despite the limitations of the ORBITA study, it highlights the importance of including a control group with a sham procedure, which would be highly desirable in CTO studies as well. The "Sham-controlled Intervention to Improve QOL in CTOs"-trial (SHINE-CTO) will hopefully provide clarification in this regard.

The present patient cohort was only treated with beta-blockers in 77.9% of cases and with other antianginal medications in only 16.2% of cases, indicating suboptimal antianginal therapy. However, it should be noted that this assessment of medication was conducted at the time of PCI, without considering previous or unsuccessful attempts at medical therapy. Furthermore, individual patient preferences regarding invasive therapy were taken into account on a case-by-case basis. In accordance with current recommendations, consideration should be given to CTO-PCI when conservative medical therapy fails to achieve adequate symptom control.

The aforementioned REVIVED study, wherein quality of life was assessed with the Kansas City Cardiomyopathy Questionnaire, also showed a decrease at the 24-month follow-up. In such study, the symptomatology was no longer different from that of patients with OMT alone[13]. However, patients with angina that limited their quality of life were excluded. This reaffirms the importance of determining the symptom severity before performing CTO-PCI. The SAQ can be an effective tool in this regard, as shown. The cut-off value of 85 points in the summary score should be validated in further prospective studies.

## Conclusion

This study showed that in patients with CTO of a coronary artery and proven viability, percutaneous revascularisation could influence the myocardial function. More clearly, however, is the impact on anginal symptoms and quality of life, which can be improved even two years after coronary intervention in most of the patients. Patients with relevant symptom severity benefit most from the procedure. Accurate assessment, such as using the SAQ and distinct cut-off values, can improve patient selection and prevent interventions without significant impact on symptoms. No other clinical or imaging parameter had this effect in the patient preselection.

### Limitations

The single-centre design and limited number of cases of this study were its clear limitations. However, the main weakness of this study was the lack of a control group. In addition, a further differentiation of the cohort according to the degree of transmurality would have been desirable. On the one hand, this was due to the number of cases and, on the other hand, not possible in a reasonable way in view of the use of different vendors and field strengths.

## Data Availability

The datasets used and/or analysed during this study are available from the corresponding author upon reasonable request.

## Abbreviations

CMR: Cardiovascular magnetic resonance imaging
CTO: Chronic total occlusion
LGE: Late gadolinium enhancement
PCI: Percutaneous coronary intervention
SAQ: Seattle Angina Questionnaire

## References

[1] Werner GS, Gitt AK, Zeymer U, Juenger C, Towae F, Wienbergen H, Senges J (2009). Chronic total coronary occlusions in patients with stable angina pectoris: impact on therapy and outcome in present day clinical practice. Clin Res Cardiol.

[2] Hoebers LP, Claessen BE, Elias J, Dangas GD, Mehran R, Henriques JPS (2015). Meta-analysis on the impact of percutaneous coronary intervention of chronic total occlusions on left ventricular function and clinical outcome. Int J Cardiol.

[3] Nakachi T, Kato S, Kirigaya H, Iinuma N, Fukui K, Saito N, Iwasawa T, Kosuge M, Kimura K, Tamura K (2017). Prediction of functional recovery after percutaneous coronary revascularization for chronic total occlusion using late gadolinium enhanced magnetic resonance imaging. J Cardiol.

[4] Tomasello SD, Boukhris M, Giubilato S, Marzà F, Garbo R, Contegiacomo G, Marzocchi A, Niccoli G, Gagnor A, Varbella F, Desideri A, Rubartelli P, Cioppa A, Baralis G, Galassi AR (2015). Management strategies in patients affected by chronic total occlusions: results from the Italian Registry of Chronic Total Occlusions. Eur Heart J.

[5] Neumann F-J, Sousa-Uva M, Ahlsson A, Alfonso F, Banning AP, Benedetto U, Byrne RA, Collet J-P, Falk V, Head SJ, Jüni P, Kastrati A, Koller A, Kristensen SD, Niebauer J, Richter DJ, Seferovic PM, Sibbing D, Stefanini GG, Windecker S, Yadav R, Zembala MO (2019). 2018 ESC/EACTS guidelines on myocardial revascularization. Eur Heart J.

[6] Kersten J, Eberhardt N, Prasad V, Keßler M, Markovic S, Mörike J, Nita N, Stephan T, Tadic M, Tesfay T, Rottbauer W, Buckert D (2021). Non-invasive imaging in patients with chronic total occlusions of the coronary arteries-what does the interventionalist need for success?. Front Cardiovasc Med.

[7] Bucciarelli-Ducci C, Auger D, Di Mario C, Locca D, Petryka J, O'Hanlon R, Grasso A, Wright C, Symmonds K, Wage R, Asimacopoulos E, Del Furia F, Lyne JC, Gatehouse PD, Fox KM, Pennell DJ (2016). CMR guidance for recanalization of coronary chronic total occlusion. JACC Cardiovasc Imaging.

[8] Spertus JA, Winder JA, Dewhurst TA, Deyo RA, Prodzinski J, McDonell M, Fihn SD (1995). Development and evaluation of the Seattle Angina Questionnaire: a new functional status measure for coronary artery disease. J Am Coll Cardiol.

[9] Baks T, van Geuns R-J, Duncker DJ, Cademartiri F, Mollet NR, Krestin GP, Serruys PW, de Feyter PJ (2006). Prediction of left ventricular function after drug-eluting stent implantation for chronic total coronary occlusions. J Am Coll Cardiol.

[10] Galassi AR, Boukhris M, Toma A, Elhadj ZI, Laroussi L, Gaemperli O, Behnes M, Akin I, Lüscher TF, Neumann FJ, Mashayekhi K (2017). Percutaneous coronary intervention of chronic total occlusions in patients with low left ventricular ejection fraction. JACC Cardiovasc Interv.

[11] Adachi H, Asanuma T, Masuda K, Nakatani S (2020). Deterioration of longitudinal, circumferential, and radial myocardial strains during acute coronary flow reduction: which direction of strain should be analyzed for early detection?. Int J Cardiovasc Imaging.

[12] Zhang L, Tian J, Yang X, Liu J, He Y, Song X (2022). Quantification of strain analysis and late gadolinium enhancement in coronary chronic total occlusion: a cardiovascular magnetic resonance imaging follow-up study. Quant Imaging Med Surg.

[13] Perera D, Clayton T, O'Kane PD, Greenwood JP, Weerackody R, Ryan M, Morgan HP, Dodd M, Evans R, Canter R, Arnold S, Dixon LJ, Edwards RJ, de Silva K, Spratt JC, Conway D, Cotton J, McEntegart M, Chiribiri A, Saramago P, Gershlick A, Shah AM, Clark AL, Petrie MC (2022). Percutaneous revascularization for ischemic left ventricular dysfunction. N Engl J Med.

[14] Stone GW on behalf of the ISCHEMIA Research Group (2021) Impact of completeness of revascularization on clinical outcomes with stable ischemic heart disease treated with an invasive versus conservative strategy: the ISCHEMIA trial

[15] Puyol-Ruiz F, Chueca-González EM, Carrasco-Chinchilla F, López-Benítez JL, Alonso-Briales JH, Melero-Tejedor JM, Hernández-García, Jiménez-Navarro M (2022). Clinical impact of complete revascularization on real-life diabetic patients. RECICE.

[16] Borgia F, Viceconte N, Ali O, Stuart-Buttle C, Saraswathyamma A, Parisi R, Mirabella F, Dimopoulos K, Di Mario C (2012). Improved cardiac survival, freedom from MACE and angina-related quality of life after successful percutaneous recanalization of coronary artery chronic total occlusions. Int J Cardiol.

[17] Werner GS, Martin-Yuste V, Hildick-Smith D, Boudou N, Sianos G, Gelev V, Rumoroso JR, Erglis A, Christiansen EH, Escaned J, Di Mario C, Hovasse T, Teruel L, Bufe A, Lauer B, Bogaerts K, Goicolea J, Spratt JC, Gershlick AH, Galassi AR, Louvard Y (2018). A randomized multicentre trial to compare revascularization with optimal medical therapy for the treatment of chronic total coronary occlusions. Eur Heart J.

[18] Brilakis ES, Mashayekhi K, Tsuchikane E, Abi Rafeh N, Alaswad K, Araya M, Avran A, Azzalini L, Babunashvili AM, Bayani B, Bhindi R, Boudou N, Boukhris M, Božinović NŽ, Bryniarski L, Bufe A, Buller CE, Burke MN, Büttner HJ, Cardoso P, Carlino M, Christiansen EH, Colombo A, Croce K, de Los D, Santos F, de Martini T, Dens J, Di Mario C, Dou K, Egred M, ElGuindy AM, Escaned J, Furkalo S, Gagnor A, Galassi AR, Garbo R, Ge J, Goel PK, Goktekin O, Grancini L, Grantham JA, Hanratty C, Harb S, Harding SA, Henriques JPS, Hill JM, Jaffer FA, Jang Y, Jussila R, Kalnins A, Kalyanasundaram A, Kandzari DE, Kao H-L, Karmpaliotis D, Kassem HH, Knaapen P, Kornowski R, Krestyaninov O, Kumar AVG, Laanmets P, Lamelas P, Lee S-W, Lefevre T, Li Y, Lim S-T, Lo S, Lombardi W, McEntegart M, Munawar M, Navarro Lecaro JA, Ngo HM, Nicholson W, Olivecrona GK, Padilla L, Postu M, Quadros A, Quesada FH, Prakasa Rao VS, Reifart N, Saghatelyan M, Santiago R, Sianos G, Smith E, Spratt C, J, Stone GW, Strange JW, Tammam K, Ungi I, Vo M, Vu VH, Walsh S, Werner GS, Wollmuth JR, Wu EB, Wyman RM, Xu B, Yamane M, Ybarra LF, Yeh RW, Zhang Q, Rinfret S, (2019). Guiding principles for chronic total occlusion percutaneous coronary intervention. Circulation.

[19] Al-Lamee R, Thompson D, Dehbi H-M, Sen S, Tang K, Davies J, Keeble T, Mielewczik M, Kaprielian R, Malik IS, Nijjer SS, Petraco R, Cook C, Ahmad Y, Howard J, Baker C, Sharp A, Gerber R, Talwar S, Assomull R, Mayet J, Wensel R, Collier D, Shun-Shin M, Thom SA, Davies JE, Francis DP (2018). Percutaneous coronary intervention in stable angina (ORBITA): a double-blind, randomised controlled trial. Lancet.

